# Compression Testing of High-Performance Carbon Fiber Composites Using Cross-Ply Laminates: A Multi-Scale Investigation of the In Situ Effect

**DOI:** 10.3390/ma19102114

**Published:** 2026-05-18

**Authors:** Xiaolong Li, Minge Duan, Jiahui Xie, Lei Li, Guangqi Huang, Guibin Song

**Affiliations:** National Key Laboratory of Strength and Structural Integrity, Aircraft Strength Research Institute of China, Xi’an 710065, China; lixl165@avic.com (X.L.); xjhkjb623@163.com (J.X.); lilei@cae.ac.cn (L.L.); huangguangqi234@sina.com (G.H.); songgb001@avic.com (G.S.)

**Keywords:** cross-ply laminates, high-performance carbon fiber composites, in situ effect, failure mechanisms, multi-scale analysis

## Abstract

Compression testing of high-performance carbon fiber composites remains challenging due to premature failure modes in unidirectional laminates, which can underestimate true material strength. This study investigates the compressive behavior of T800-grade carbon fiber-reinforced polymer (CFRP) cross-ply ([90/0]_2s_) and unidirectional ([0]_8_) laminates using finite element simulation and experimental testing following the SACMA SRM-1R-94 standard, combined with macroscopic and microscopic failure analysis. The results show that cross-ply laminates consistently exhibit valid mid-gauge failure with lower data dispersion (coefficient of variation: 3.44%), whereas unidirectional laminates are prone to invalid root failures (crushing or shear). The compressive strength derived from cross-ply laminates using the back-out factor (2040 MPa) is 13% higher than that from direct unidirectional testing (1802 MPa), attributed to the in situ effect where adjacent 90-degree plies suppress fiber microbuckling. The cross-ply approach provides a more reliable and practical method for characterizing the true in situ compressive strength of high-performance CFRP composites.

## 1. Introduction

Carbon fiber-reinforced polymer (CFRP) composites have been widely used in aerospace, rail transit, wind turbine blades, and marine engineering due to their high specific strength, high specific modulus, excellent fatigue resistance, and design flexibility [1,2]. With the continuous advancement of carbon fiber technology, from early T300-grade to current T800- and T1100-grade high-performance fibers, the mechanical properties of composites have significantly improved, posing new challenges to traditional mechanical characterization methods [3,4,5].

Compressive performance is a critical indicator in the structural design of composites, especially in load-bearing components such as fuselages, wings, and wind turbine blades [6,7,8]. Compared with tensile properties, compressive performance is more sensitive to matrix properties, fiber–matrix interface quality, and initial fiber defects, with more complex failure mechanisms [9,10]. Studies have shown that compressive failure in fiber-reinforced plastics typically involves instabilities at multiple scales, including single-fiber microbuckling, kink-band formation in fiber bundles, and global buckling of laminates [11,12,13]. These complex failure mechanisms make accurate characterization of compressive performance a key challenge in composite mechanics research [14,15].

Currently, commonly used international standards for the compressive testing of composites include ASTM D695, ASTM D3410, SACMA SRM-1R-94, and ASTM D6641 [16,17,18,19]. These standards can be classified into end-loading, shear-loading, and combined loading methods [20]. ASTM D695 uses end-loading and is suitable for rigid plastics and various composites, but its application to high-modulus, high-strength composites is limited [16]. ASTM D3410 uses shear-loading through wedge grips, effectively avoiding end crushing, but requires high specimen machining precision and fixture quality [17]. SACMA SRM-1R-94 uses end-loading with tabs to transfer loads, making it suitable for oriented continuous high-modulus fiber-reinforced composites; its end-support design reduces shear interference and simplifies operation [19,20]. ASTM D6641 combines end- and shear-loading and is currently one of the most widely used standards [21,22].

However, most of these standards were developed in the 1980s and 1990s, primarily for T300-grade and lower composites [23]. When applied to T800-grade and higher high-strength, high-modulus carbon fiber composites, the significantly increased compressive failure load in the same layup ([0]_8_) imposes higher demands on tab bonding strength and specimen machining precision [3,24]. Some comparative studies have shown that SACMA SRM-1R-94 is prone to invalid failure modes such as crushing-induced tab failure (CIT) or slanted gauge section shear failure (SGV) in high-strength, high-modulus carbon fiber composites, often yielding compressive strengths lower than the true material values [25]. Small machining errors or bonding defects can induce stress concentration and premature failure, hindering material development and precise structural design.

Existing standards typically use unidirectional laminates ([0]_ns_) to obtain compressive properties and treat the measured strength as an intrinsic property for failure analysis [26,27]. However, Dvorak et al. found that the strength of a ply in a multidirectional laminate differs significantly from that in a unidirectional laminate [28]. This “in situ effect” suggests that the actual strength of a ply in a laminate, influenced by adjacent plies, can be several times that of a unidirectional laminate [29,30]. The in situ strength is not constant but depends on factors such as ply thickness, adjacent ply orientation, and stacking sequence [31,32,33]. This finding challenges traditional composite strength analysis, as using unidirectional ply properties for multidirectional laminates may lead to conservative design or inaccurate failure prediction [34,35].

To address this issue, some researchers have proposed using cross-ply laminates ([90/0]_ns_; in the laminate notation used throughout this paper, the subscript ‘s’ denotes a symmetric laminate, and the subscript ‘ns’ in [90/0]_ns_ indicates n repetitions of the [90/0] sequence followed by a symmetric mirror) for compression tests and deriving the unidirectional compressive strength using a “back-out factor” (BF) based on classical lamination theory [36,37,38]. However, the failure mechanisms of high-performance CFRP cross-ply laminates under compression are not yet fully understood, and systematic comparisons involving simulation, experimentation, and micro-mechanism analysis are lacking. The validity and applicability of this back-out method require further verification [39,40,41].

In this context, this study conducts a comparative compression test on unidirectional and cross-ply T800-grade CFRP laminates. First, finite element models are developed in ABAQUS using the Hashin failure criterion to analyze stress distribution and failure characteristics. Second, compression tests following SACMA SRM-1R-94 are performed to compare failure modes and strength. The back-out factor is derived from classical lamination theory, and the unidirectional compressive strength is estimated from cross-ply test results. Finally, macroscopic and microscopic failure mechanisms are analyzed using optical microscopy and scanning electron microscopy (SEM) to reveal failure mechanism differences and validate the cross-ply back-out method. This study aims to provide theoretical and technical support for accurate compressive characterization and standard improvement of high-performance CFRP.

## 2. Computational Analysis of Compressive Behavior

### 2.1. Model Setup

Finite element models were built in ABAQUS following the SACMA SRM-1R-94 specimen geometry, with layups of [0]_8_ ([0/0/0/0/0/0/0/0]) and [90/0]_2s_ ([90/0/90/0/0/90/0/90]). The composite material was modeled using the Lamina constitutive model with the Hashin failure criterion. The Hashin failure criterion is employed here to predict failure initiation and approximate failure patterns; however, it does not fully capture progressive damage mechanisms such as kink-band evolution and fiber microbuckling propagation. Input material parameters are listed in Table 1. The SC8R shell elements were adopted for the gauge section of the specimen. To balance computational accuracy and efficiency, mesh refinement was applied to the gauge section, which contains a total of 2000 elements. Boundary conditions included fixed support at one end and 1 mm displacement at the other.

The central elements at the cross-section of the gauge section were selected to extract the average stress–strain curves. The modulus of the linear segment was calculated as 155.16 GPa, with a deviation of less than 1% from the input value, which verifies the accuracy of the simulation results.

### 2.2. Simulation Results

**Figure 1:**
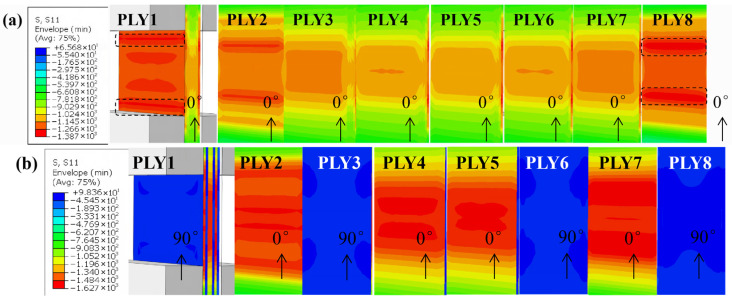
Longitudinal stress distribution in (**a**) unidirectional and (**b**) cross-ply laminates.

Figure 1 shows the stress distribution prior to failure. For the unidirectional laminates (Figure 1a), stress is concentrated in the gauge section, with significant stress concentration near the tab–gauge transition, as marked by the black box in the figure, potentially inducing premature damage. For the cross-ply laminates (Figure 1b), longitudinal compressive stress was concentrated in the 0-degree plies, while 90-degree plies carried negligible stress. The cross-ply design distributed the transition effect, reducing stress concentration at the tab root and promoting failure within the gauge section.

**Figure 2:**
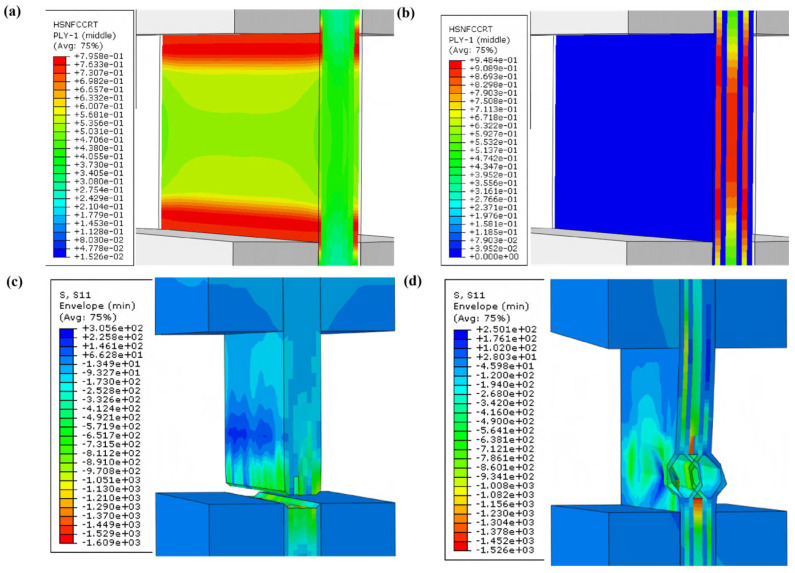
Fiber compression failure index distribution: (**a**) unidirectional before failure; (**b**) cross-ply before failure; (**c**) unidirectional failure mode; (**d**) cross-ply failure mode.

Hashin fiber compression failure index (Figure 2) showed that in unidirectional laminates, maximum damage occurred at the tab–gauge interface (Figure 2a), leading to root failure (Figure 2c). In cross-ply laminates, damage was concentrated in the mid-gauge region of 0-degree plies (Figure 2b), resulting in mid-section failure (Figure 2d).

The simulation indicates that unidirectional laminates are prone to premature root failure due to stress concentration, while cross-ply laminates promote valid mid-section failure.

## 3. Materials and Methods

### 3.1. Materials

T800 CFRP prepreg (manufactured by AVIC Composite, Beijing, China) was used, with a cured ply thickness of 0.125 mm. Two laminates were fabricated: unidirectional [0]_8_ and cross-ply [90/0]_2s_. Specimen geometry followed SACMA SRM-1R-94, with a nominal gauge thickness of 1 mm and gauge length of 4.75 mm.

### 3.2. Testing Procedure

**Figure 3:**
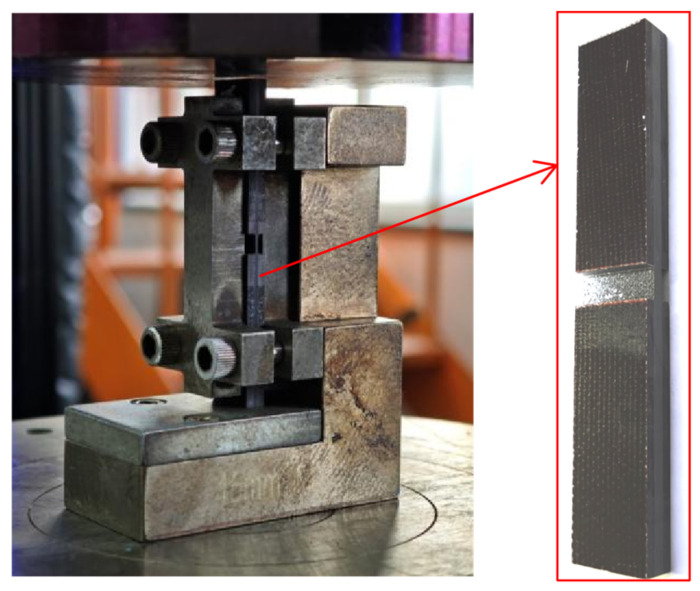
Compression test state diagram.

Compression tests were performed using a SACMA SRM-1R-94 fixture with a torque of 1 N·m. The fixture was mounted on a universal testing machine (manufactured by LSI system, Shanghai, China), and specimens were loaded at 1 mm/min until failure (Figure 3).

Potential sources of data scatter—including specimen misalignment in the fixture, variations in tab adhesive thickness, and tab bonding quality—were minimized where possible. All specimens were visually inspected for adhesive voids, and alignment was verified using a precision jig. The tightening torque of the SACMA fixture was kept constant at 1 N·m. Despite these precautions, some scatter inherent to compression testing of high-strength composites could not be eliminated, as reflected in the CV values.

### 3.3. Data Reduction

Compressive strength for unidirectional laminates was calculated as:


(1) $$\sigma_{xc}=\frac{P_{\max}}{wt}$$

The classical laminate theory is a material mechanics theory based on the strain and deformation of laminates. On the basis of this theory, the relationship between a single ply and the laminate can be established. Therefore, using the classical laminate theory, the unidirectional compressive strength can be derived from the compression test results of orthogonally layered composite laminates. The formula for calculating the unidirectional ply compressive strength of orthogonally layered composites is given by Equation (2).


(2) $${\left(\sigma_{x}\right)}_{0}=BF\frac{P_{\max}}{wt}$$

BF is the back-out factor, where BF=12E1(E1+E2)−(V12E2)214(E1+E2)2−(V12E2)2 for the [90/0]_2s_ layup [42]. In this study, the BF value was calculated to be 1.865 based on the fundamental material parameters provided by the manufacturer.

Physically, the back-out factor (BF) represents the ratio of the longitudinal stress carried by the 0° plies to the average laminate stress in a ([90/0]_2s_) laminate. It accounts for the load sharing between the 0° and 90° layers and the effects of Poisson’s mismatch, thereby allowing the intrinsic 0–ply compressive strength to be extracted from the measured laminate strength.

## 4. Results

### 4.1. Experimental Results

Test results are summarized in Table 2. The normalized unidirectional compressive strength from direct testing was 1802 MPa, while the cross-ply-derived value was 2040 MPa, showing a difference of approximately 13%. The cross-ply results exhibited lower dispersion (CV: 3.44%) compared to unidirectional results (CV: 6.57%).

### 4.2. Failure Mode Analysis

**Figure 4:**
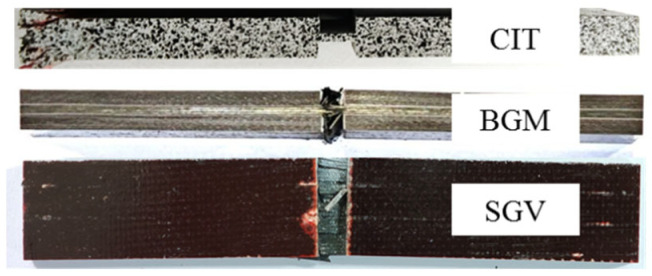
Failure mode of the compression test.

Unidirectional laminates exhibited multiple failure modes, including CIT (end crushing), SGV (slanted shear failure), and BGM (mid-section burst), as shown in Figure 4. CIT was considered invalid. In contrast, all cross-ply specimens failed in BGM mode, which is acceptable per the standard. The average failure load for cross-ply specimens was approximately 60% that of unidirectional specimens, reducing demands on tab bonding quality.

In the finite element simulation of unidirectional laminates, significant stress concentration occurs at the tab–gauge transition, and initial damage emerges at the contact interface between tabs and the gauge section, which is consistent with the SGV failure mode observed in experiments. For cross-ply laminates, the finite element simulation presents an open-notch failure characteristic, which is in full agreement with the BGM failure mode in the tests. This agreement confirms that the simulation captures the critical features that govern the validity of the experimental failure modes.

### 4.3. Macroscopic Fracture Analysis

**Figure 5:**
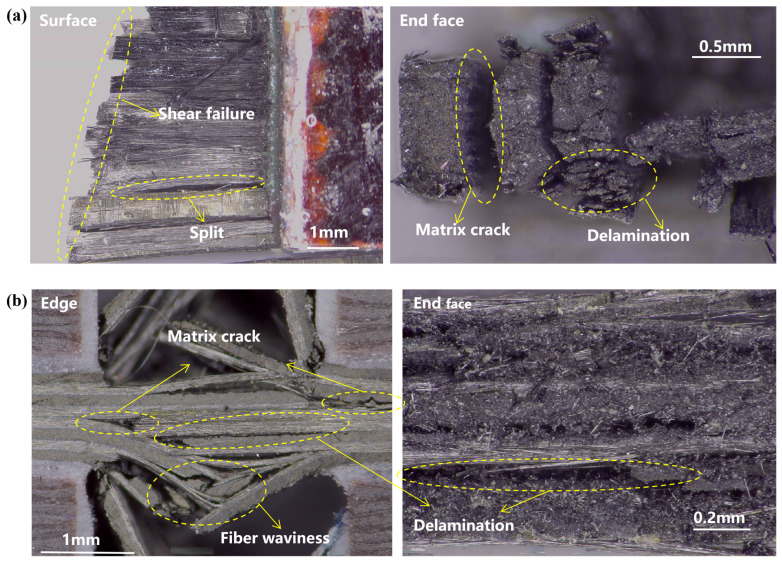
Macroscopic fracture morphology: (**a**) unidirectional laminates; (**b**) cross-ply laminates.

Optical microscopy (Figure 5) revealed that unidirectional laminates failed with longitudinal splitting and oblique shear fracture, while cross-ply laminates exhibited explosive failure with distinct ply separation and fiber breakage in 0-degree plies and matrix cracking in 90-degree plies. The 90-degree plies effectively suppressed fiber microbuckling in the 0-degree plies.

### 4.4. Microscopic Fracture Analysis

**Figure 6:**
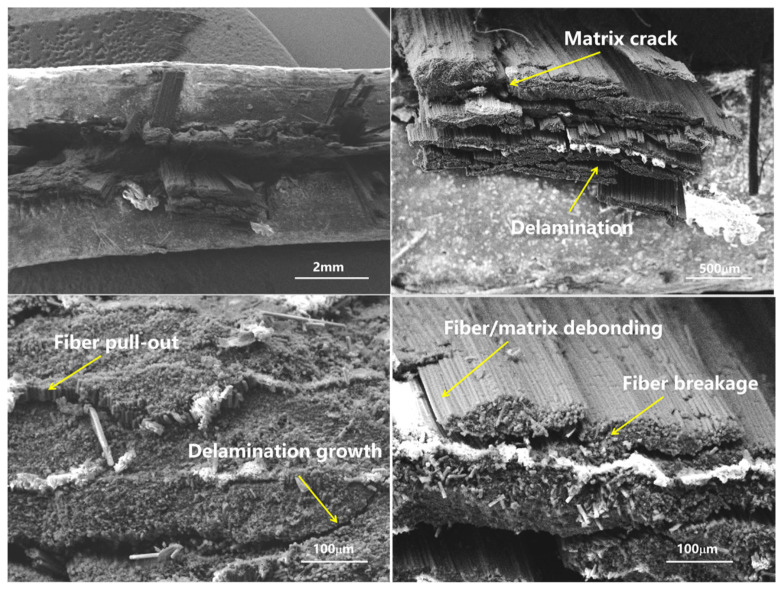
Microscopic fracture morphology of unidirectional laminate compression failure.

**Figure 7:**
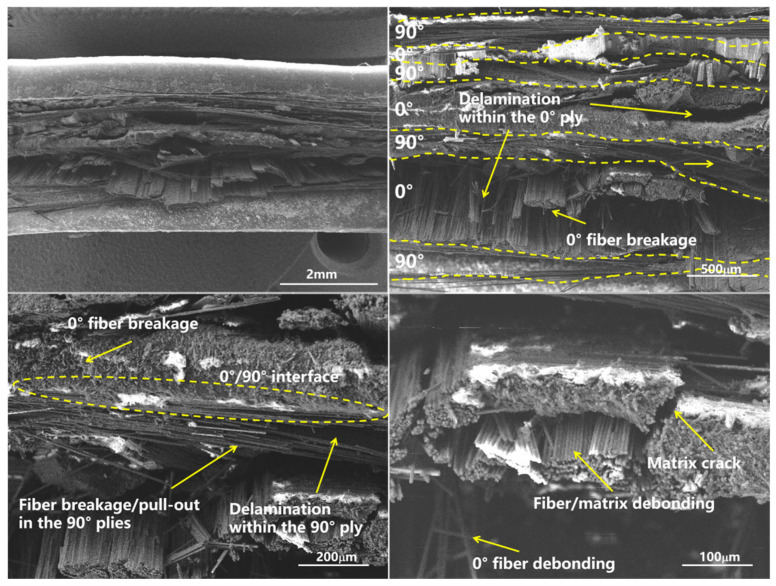
Microscopic fracture morphology of cross-ply laminate compression failure.

SEM observations (Figure 6 and Figure 7) showed that both laminates exhibited matrix cracking, fiber breakage, fiber–matrix debonding, and delamination. In unidirectional laminates, fiber microbuckling dominated, with extensive fiber pull-out and smooth fiber surfaces. In cross-ply laminates, the 0-degree plies showed well-aligned fiber fractures, while the 90-degree plies exhibited transverse cracks and fiber pull-out. The interface between 0-degree and 90-degree plies showed evidence of constrained failure.

## 5. Discussion

The present study provides a comprehensive comparison of the compressive behavior of unidirectional and cross-ply T800 CFRP laminates through integrated finite element simulation and experimental testing. The results demonstrate that the cross-ply configuration significantly improves the validity and reliability of compression testing for high-performance composites, a finding with important implications for both standardization and practical structural design.

### 5.1. Validity of Failure Modes

The simulation results in Section 2.2 clearly indicate that unidirectional laminates suffer from pronounced stress concentration at the tab–gauge interface, which predisposes them to premature root failure (CIT or SGV). In contrast, the cross-ply laminates effectively mitigate this issue by introducing 90-degree plies at the surfaces, which act as a transitional layer that reduces the stress gradient near the tabs. This structural modification shifts the failure initiation site to the mid-gauge region, as evidenced by both the simulated damage distribution (Figure 2b) and the experimental failure modes (Section 4.2). The BGM failure mode observed in all cross-ply specimens is considered valid per SACMA SRM-1R-94, whereas the unidirectional specimens showed a high proportion of invalid failures (CIT). This suggests that the cross-ply configuration is more tolerant to minor manufacturing imperfections and tab bonding inconsistencies, which are common sources of variability in composite testing [25,36].

### 5.2. Implications of the In Situ Effect

The higher and less variable compressive strength obtained from cross-ply laminates (2040 MPa vs. 1802 MPa) can be attributed to the in situ effect [28,29,30], whereby the adjacent 90° plies provide lateral constraint that suppresses fiber microbuckling—the dominant compressive failure mechanism [9,11]. This constraint effect is analogous to the in situ strengthening phenomenon observed in transverse tension and shear [29,31]. This interpretation is directly supported by the microscopic observations (Section 4.4): unidirectional laminates exhibited extensive fiber pull-out and smooth fiber surfaces, indicating uncontrolled microbuckling, whereas cross-ply laminates showed aligned fiber fractures with minimal pull-out, confirming effective lateral support from the 90° plies. Hence, the compressive strength derived from cross-ply laminates may more faithfully represent the actual load-bearing capacity of 0° plies in a multidirectional laminate, in line with their intended use in structural applications [33,34].

### 5.3. Practical Advantages of the Cross-Ply Approach

From a practical perspective, the reduced failure load (approximately 60% of that for unidirectional laminates) and the stable BGM failure mode make cross-ply testing more robust. This reduced load requirement eases demands on tab bonding quality, specimen machining, and fixture alignment, thereby lowering the entry barrier for reliable compression testing in research and quality control settings. Moreover, the lower dispersion (CV: 3.44% vs. 6.57%) enhances statistical confidence, which is critical for material qualification and the development of design allowables.

### 5.4. Comparison with Previous Studies

The BF value of 1.865 calculated in this study is consistent with theoretical predictions and falls within the range reported in the literature for similar material systems [36,38,42]. The derived unidirectional strength of 2040 MPa is higher than the directly measured value (1802 MPa) but is comparable to the manufacturer’s reported compressive strength (approximately 2000 MPa) for T800/epoxy systems when tested under optimized conditions. This supports the assertion that direct unidirectional testing may underestimate the true in situ strength due to premature failure induced by stress concentrations and manufacturing defects.

### 5.5. Limitations and Future Work

Despite the clear advantages, several limitations should be acknowledged. First, the study focuses on a single material system (T800/epoxy) and a specific cross-ply laminate ([90/0]_2s_). The generalizability of the BF approach to other fiber types, matrix systems, and stacking sequences requires further validation. Second, the present analysis is limited to quasi-static loading; the behavior under fatigue or dynamic loading may differ and warrants investigation. Third, while the Hashin failure criterion effectively captured the failure initiation trends, it does not account for progressive damage or the post-failure behavior, which may be relevant for certain design scenarios. Future work should explore these aspects, potentially incorporating cohesive zone models or continuum damage mechanics approaches.

## 6. Conclusions

This study systematically compared the compressive behavior of unidirectional and cross-ply T800 CFRP laminates through finite element simulation, experimental testing, and microscopic analysis. The following conclusions are drawn:

1. The cross-ply configuration effectively mitigates stress concentration at the tab–gauge interface, promoting valid mid-gauge failure (BGM) and significantly reducing the incidence of invalid failure modes such as CIT and SGV.
2. The cross-ply approach yields more reliable compressive strength data with lower statistical dispersion (CV: 3.44%) compared to unidirectional testing (CV: 6.57%), enhancing test reproducibility.
3. The failure load for cross-ply laminates is approximately 60% of that for unidirectional laminates, reducing demands on tab bonding quality and fixture alignment.
4. The higher compressive strength derived from cross-ply laminates (2040 MPa) compared to direct unidirectional testing (1802 MPa) is attributed to the in situ effect, where adjacent 90-degree plies constrain fiber microbuckling and delay failure.
5. The cross-ply testing method, combined with the back-out factor derived from classical lamination theory, offers a robust and practical alternative for characterizing the true in situ compressive strength of high-performance CFRP composites.

It should be noted that this approach depends on the accurate determination of the back-out factor and has so far been validated only for [90/0]_ns_ layups. Further evaluation on different stacking sequences and material systems is advisable to establish its broader applicability.

## Figures and Tables

**Table 1:** Material properties used in FEM.

E_1_/MPa	E_2_/Mpa	υ_12_	G_12_ = G_13_/Mpa	G_23_/Mpa
155,000	11,300	0.28	6580	4414
**X_T_/MPa**	**X_C_/MPa**	**Y_T_/Mpa**	**Y_C_/Mpa**	**S/Mpa**
3320	1650	68.7	280	73.7

In Table 1, E_1_ and E_2_ denote Young’s moduli in the fiber and transverse directions, respectively; G_12_, G_13_, and G_23_ are the shear moduli; υ_12_ is Poisson’s ratio; X_T_ and X_C_ represent the longitudinal tensile and compressive strengths; Y_T_ and Y_C_ denote the transverse tensile and compressive strengths; and S is the shear strength.

**Table 2:** Compressive test results.

Lamination Type	Specimen Number	Load (kN)	σ_0c_ (MPa)		Failure Mode
			Measured Value	Regularization Value	
unidirectional ply	[0]_8_-1 *	24.2	1969 *	1910 *	CIT
	[0]_8_-2	24.8	2129	1959	BGM
	[0]_8_-3	23.9	1864	1883	SGV
	[0]_8_-4	22.1	1871	1740	SGV
	[0]_8_-5	21.1	1786	1661	SGV
	[0]_8_-6	22.4	1842	1768	SGV
	Average Value		1898	1802	—
	Standard Deviation		133	118	—
	CV(%)		7.03	6.57	—
cross-ply	[90/0]_2s_-1	13.7	2057	1996	BGM
	[90/0]_2s_-2	14.1	2130	2066	BGM
	[90/0]_2s_-3	14.5	2214	2126	BGM
	[90/0]_2s_-4	13.6	2097	1992	BGM
	[90/0]_2s_-5	14.4	2195	2108	BGM
	[90/0]_2s_-6	13.3	2077	1952	BGM
	Average Value		2129	2040	—
	Standard Deviation		64.1	70.1	—
	CV(%)		3.01	3.44	—

Note: Test failure modes marked with * are invalid and their results are not included in statistics.

## Data Availability

The original contributions presented in this study are included in the article. Further inquiries can be directed to the corresponding author.

## Abbreviation

The following abbreviation is used in this manuscript:

BF	Back-out Factor

## References

[1] Tiwary A., Kumar R., Chohan J.S. (2022). A review on characteristics of composite and advanced materials used for aerospace applications. Mater. Today Proc..

[2] Zhang X., Chen Y., Hu J. (2018). Recent advances in the development of aerospace materials. Prog. Aerosp. Sci..

[3] Lu Z., Zeng J., Liu X. (2022). Research and Application Progress of T1000 Grade Carbon Fiber and Composite Materials. Aerosp. Manuf. Technol..

[4] Nunna S., Ravindran A.R., Mroszczok J., Creighton C., Varley R.J. (2023). A review of the structural factors which control compression in carbon fibres and their composites. Compos. Struct..

[5] Newcomb B.A. (2016). Processing, structure, and properties of carbon fibers. Compos. Part A Appl. Sci. Manuf..

[6] Oya N., Johnson D.J. (2001). Longitudinal compressive behaviour and microstructure of PAN-based carbon fibres. Carbon.

[7] Jumahat A., Soutis C., Jones F.R., Hodzic A. (2010). Fracture mechanisms and failure analysis of carbon fibre/toughened epoxy composites subjected to compressive loading. Compos. Struct..

[8] Budiansky B., Fleck N.A. (1993). Compressive failure of fibre composites. J. Mech. Phys. Solids.

[9] Pinho S.T., Iannucci L., Robinson P. (2006). Physically-based failure models and criteria for laminated fibre-reinforced composites with emphasis on fibre kinking: Part I: Development. Compos. Part A Appl. Sci. Manuf..

[10] Fleck N.A. (1997). Compressive failure of fiber composites. Adv. Appl. Mech..

[11] Opelt C.V., Cândido G.M., Rezende M.C. (2018). Compressive failure of fiber reinforced polymer composites–A fractographic study of the compression failure modes. Mater. Today Commun..

[12] Krishnappa S., Gururaja S. (2024). Compressive failure mechanisms in unidirectional fiber reinforced polymer composites with embedded wrinkles. Compos. Part B Eng..

[13] Grotz L., Kirane K. (2024). Characterizing compressive failure mechanisms and their transitions in woven composites under on and off-axis loading. Compos. Struct..

[14] Waas A.M., Schultheisz C.R. (1996). Compressive failure of composites, part II: Experimental studies. Prog. Aerosp. Sci..

[15] Schultheisz C.R., Waas A.M. (1996). Compressive failure of composites, part I: Testing and micromechanical theories. Prog. Aerosp. Sci..

[16] (2023). Standard Test Method for Compressive Properties of Rigid Plastics.

[17] (2003). Standard Test Method for Compressive Properties of Polymer Matrix Composite Materials with Unsupported Gage Section by Shear Loading.

[18] (2009). Standard Test Method for Compressive Properties of Polymer Matrix Composite Materials Using a Combined Loading Compression (CLC) Test Fixture.

[19] (1994). SACMA Recommended Test Method for Compressive Properties of Oriented Fiber-Resin Composites.

[20] Li L., Yuan Z., Wang Y. (2022). A Comparative Research on Test Methods for the Compressive Properties of Unidirectional Carbon Fiber Reinforced Composites. Aerosp. Mater. Technol..

[21] Adams D.F., Welsh J.S. (1997). The Wyoming combined loading compression (CLC) test method. Compos. Technol. Res..

[22] Wegner P.M., Adams D.F. (2000). Verification of the Combined Load Compression (CLC) Test Method.

[23] Adams D.F. (1995). Current status of compression testing of composite materials. Mater. Chall. Diversif. Future.

[24] Peng G., Guo L., Shi F. (2023). Development status and suggestion of the third generation PAN-based carbon fiber. New Chem. Mater..

[25] Wei H.Y., Yang S.C., Shen Z., Zhou J.F., Shen W., Wang J. Research on Test Methods for Determining the Compressive Properties of Composite Materials. Proceedings of the 15th National Conference on Composite Materials (NCCM-15).

[26] Hashi Z. (1980). Failure criteria for unidirectional fiber composites. J. Appl. Mech..

[27] Daniel I.M., Ishai O., Daniel I.M., Daniel I. (1994). Engineering Mechanics of Composite Materials.

[28] Dvorak G.J., Laws N. (1987). Analysis of progressive matrix cracking in composite laminates II. First ply failure. J. Compos. Mater..

[29] Camanho P.P., Dávila C.G., Pinho S.T., Iannucci L., Robinson P. (2006). Prediction of in situ strengths and matrix cracking in composites under transverse tension and in-plane shear. Compos. Part A Appl. Sci. Manuf..

[30] Parvizi A., Garrett K.W., Bailey J.E. (1978). Constrained cracking in glass fibre-reinforced epoxy cross-ply laminates. J. Mater. Sci..

[31] Arteiro A., Catalanotti G., Melro A.R., Linde P., Camanho P.P. (2015). Micro-mechanical analysis of the effect of ply thickness on the transverse compressive strength of polymer composites. Compos. Part A Appl. Sci. Manuf..

[32] Chang F.K., Chen M.H. (1987). The in situ ply shear strength distributions in graphite/epoxy laminated composites. J. Compos. Mater..

[33] Flaggs D.L., Kural M.H. (1982). Experimental determination of the in situ transverse lamina strength in graphite/epoxy laminates. J. Compos. Mater..

[34] Sun C.T., Tao J. (1998). Prediction of failure envelopes and stress/strain behaviour of composite laminates. Compos. Sci. Technol..

[35] Davila C., Jaunky N., Goswami S. (2003). Failure criteria for FRP laminates in plane stress. J. Compos. Mater..

[36] Welsh J.S., Adams D.F. (1997). Testing of angle-ply laminates to obtain unidirectional composite compression strengths. Compos. Part A Appl. Sci. Manuf..

[37] Welsh J.S., Adams D.F. (2002). An experimental investigation of the biaxial strength of IM6/3501-6 carbon/epoxy cross-ply laminates using cruciform specimens. Compos. Part A Appl. Sci. Manuf..

[38] Scafè M., Labanti M., Coglitore A., Raiteri G., Dlacic R., Troiani E., Falaschetti M.P. (2013). Experimental determination of compressive strength of an unidirectional composite lamina: Indirect estimate by Using Back-out Factor (BF). Grup. Ital. Frat. Convegno Naz. IGF Acta Fract..

[39] Thomson D., Cui H. (2019). A study on the longitudinal compression strength of fibre reinforced composites under uniaxial and off-axis loads using cross-ply laminate specimens. Compos. Part A Appl. Sci. Manuf..

[40] Zou W., Tong Y., Wang Y. (2026). The influence mechanism of multi-scale structural evolution in carbon fibres on the axial compressive failure of composites. Compos. Part A Appl. Sci. Manuf..

[41] Zhang M., Wang X., Li W. (2018). Compressive strength determined for ultrahigh modulus fiber reinforced composites by [90/0] ns laminates. Compos. Struct..

[42] Li X., Song G., Xie J. (2025). Testing of Cross-Ply Laminates to Obtain Unidirectional Composite Compression Strengths. Proceedings of the 2nd Aerospace Frontiers Conference (AFC 2025).

